# Guild-based analysis for understanding gut microbiome in human health and diseases

**DOI:** 10.1186/s13073-021-00840-y

**Published:** 2021-02-09

**Authors:** Guojun Wu, Naisi Zhao, Chenhong Zhang, Yan Y. Lam, Liping Zhao

**Affiliations:** 1 Center for Nutrition, Microbiome and Health, New Jersey Institute for Food, Nutrition and Health, Rutgers University, New Brunswick, NJ USA; 2 Department of Biochemistry and Microbiology, Rutgers University, New Brunswick, NJ USA; 3 Rutgers-Jiaotong Joint Laboratory for Microbiome and Human Health, New Brunswick, NJ, USA; 4 Department of Public Health and Community Medicine, School of Medicine, Tufts University, Medford, MA USA; 5 State Key Laboratory of Microbial Metabolism, Ministry of Education Laboratory of Systems Biomedicine, Shanghai Jiao Tong University, Shanghai, China

**Keywords:** Gut microbiota, Guild, High dimensionality, High sparsity

## Abstract

To demonstrate the causative role of gut microbiome in human health and diseases, we first need to identify, via next-generation sequencing, potentially important functional members associated with specific health outcomes and disease phenotypes. However, due to the strain-level genetic complexity of the gut microbiota, microbiome datasets are highly dimensional and highly sparse in nature, making it challenging to identify putative causative agents of a particular disease phenotype. Members of an ecosystem seldomly live independently from each other. Instead, they develop local interactions and form inter-member organizations to influence the ecosystem’s higher-level patterns and functions. In the ecological study of macro-organisms, members are defined as belonging to the same “guild” if they exploit the same class of resources in a similar way or work together as a coherent functional group. Translating the concept of “guild” to the study of gut microbiota, we redefine guild as a group of bacteria that show consistent co-abundant behavior and likely to work together to contribute to the same ecological function. In this opinion article, we discuss how to use guilds as the aggregation unit to reduce dimensionality and sparsity in microbiome-wide association studies for identifying candidate gut bacteria that may causatively contribute to human health and diseases.

**Supplementary Information:**

The online version contains supplementary material available at 10.1186/s13073-021-00840-y.

## Background

In the past decade, high-throughput sequencing has revolutionized microbiome research, leading to an explosive growth of studies on the associations between gut microbiome and human diseases. These microbiome studies have linked changes in the human gut microbiome with many disease outcomes, including obesity, type 2 diabetes, liver diseases, various forms of cancer, allergies, and neurodegenerative diseases [1, 2]. However, in most cases, there is still a need to identify key gut microbial members and demonstrate their causative role in the etiology and progression of a specific disease phenotype.

Microbiome-wide association studies, powered by next-generation sequencing, have the goal of identifying candidate bacteria that may alleviate, induce, or aggravate a disease phenotype. These putative causative agents can be isolated into either a pure culture or a consortium with defined membership and then inoculated into germ-free animals to reproduce the disease phenotype. In these gnotobiotic models for human diseases, one can elucidate the molecular crosstalk between the colonizing bacteria and host to establish the molecular chain of causation between specific gut bacteria and human disease endpoints. Such bacteria and their effector molecules can become biomarkers and targets for diagnosis, prediction, treatment, and prevention of relevant diseases. Thus, identifying bacterial candidates associated with specific health outcomes and disease phenotypes is the first step for demonstrating the causative role of gut microbiome in human diseases.

However, due to the complexity and diversity of human gut microbiota, microbiome datasets are highly dimensional, low rank, and highly sparse in nature, making it very challenging to identify putative causative agents of a particular disease phenotype. High dimensionality and low rank refer to a large number of variables for a smaller number of samples. While in a highly sparse dataset, many variables are zeros in most samples. These two characteristics of microbiome dataset are primarily a consequence of the high strain-level diversity of the human microbiome. Strains refer to populations that are the non-dividable and basic building blocks in microbial ecosystems, often recognized by isolation or sequencing [3]. The human gut microbiota has been shown to have an exceedingly high strain-level diversity among individuals. For instance, based on the 1590 gut metagenomic samples from nine human-associated datasets, Truong et al. [4] found that, on average, only 35.31% species were shared between two unrelated individuals. Even among individuals whose guts were colonized by the same species, only 3.67% of the strains were common [4].

When the dimension of a highly sparse microbiome dataset increases, it becomes substantially more difficult to identify patterns relevant to specific health outcomes and disease phenotypes. At present, two customary approaches, a taxon-based method and a gene-centric method, are frequently used to reduce the dimensionality and sparsity of a microbiome dataset. While the current popular methods effectively reduce data dimensionality and sparsity, they often heavily depend on prior knowledge and aggregate bacterial members into functionally heterogeneous groups, thus often leading to controversial results. Here we propose a more ecologically relevant aggregation method that collapses individual microbiome members into ecological functional units, namely “guilds,” providing a more ecologically sound approach for the search of putative causative agents to human disease phenotypes in the gut microbiota.

### Microbiome analysis pitfalls in taxon-based analysis

Currently, a common strategy for reducing dimensionality is to collapse bacterial strains based on nearest neighbor taxonomy [5, 6], which assigns a microbiome sequence to a taxon if the sequence’s similarity to a known bacterium passes a certain threshold. The higher the taxon level bacteria are collapsed into, the lower dimensionality and sparsity one can achieve. Such analysis has been commonly used in microbiome-wide association studies [7–10], in which sequences representing individual microbial populations are collapsed into different taxonomic levels from “species,” “genus,” “family,” all the way up to “phylum.” However, correlating these taxa with disease phenotypes to derive microbiome biomarkers can often lead to controversial results.

For example, in the study of gut microbiome and obesity, collapsing data to “phylum” level had generated a decade-long debate over controversial results of the relationship between obesity and the ratio of phyla Firmicutes/Bacteroidetes (F/B ratio) [11, 12]. Many studies showed that the F/B ratio was positively associated with obesity, while other studies found no such relationship or even an opposite trend [13–15]. Two meta-analyses [9, 16], based on datasets from 11 studies, concluded that there was no consistent difference in F/B ratio between non-obese and obese individuals. It became apparent that while numerous differences existed at finer resolutions between the lean and obese human gut microbiota, these differences were not observed at higher taxonomic levels such as phylum [16]. Even at the lowest taxonomy level—“species” similar conflicting findings in microbial signatures of the same disease have been reported, e.g., the substantial controversy regarding the abundance of *Escherichia coli* in the colonic mucosa of ulcerative colitis patients [17].

One potential cause for such controversial, sometimes spurious and misleading results from the taxon-based analysis is the assumption that all members in the same taxon have the same relationship with a particular disease phenotype. While in reality, bacterial strains in the same taxonomic group have been found to vary in their relationships with the host bio-clinical parameters, suggesting that they each may have a distinct impact on host health [18, 19]. By definition, a bacterial species is the collection of “strains with approximately 70% or greater DNA-DNA relatedness” [20]. For years, species identification and classification were conducted by DNA-DNA hybridization following this definition. When DNA sequencing became widely available, microbiologists found that the 70% DNA-DNA hybridization value recommended for species identification corresponded to 95% average nucleotide identity (ANI) and 69% conserved DNA [21]. At present, species identification can be done by genomic sequencing. Members (strains) in the same bacterial species should have higher than 70% genomic homology (95% ANI), roughly equivalent to 97% homology between 16S rRNA genes. This standard means that some species could be more genetically heterogeneous than others and that two strains belonging to the same bacterial species could have up to 30% difference in their genomic makeup. Due to this high genetic diversity within a species, strains in the same species can show contrasting phenotypes, e.g., virulent or non-virulent, positively or negatively responding to high fiber supplementation [22]. This strain-level genetic and functional diversity is critical to understanding the host-bacteria symbiosis in health and diseases. For example, the variation in the capsular polysaccharide biosynthesis loci in *Bacteroides thetaiotaomicron* strains contributed to their fitness in the host gut [23], and the difference between *Bifidobacterium breve* strains in their ability to utilize carbon source is key to their adoption in the gut ecosystem [24]. If members in a taxon have opposite associations with the same disease phenotype, lumping them into one taxon variable will produce a null or a spurious correlation with the disease phenotype.

Another critical limitation of taxon-based analysis is that it often excludes novel bacteria from disease association studies. At present, the generally used practice in taxon-based analysis is assigning bacterial DNA sequence taxonomic names based on their similarity to the nearest neighbor recorded in a reference database [5, 6]. In this practice, one will categorize all sequences that do not meet the similarity cut-off to sequences with known taxonomy as unclassified, and in most cases, unclassified sequences will be left out of the subsequent taxon-based data analysis. This practice limits analysis of microbiome data to what is known and available in existing databases.

### Microbiome analysis pitfalls in gene-centric analysis

Compared with target region sequencing, e.g., hypervariable regions in the 16S rRNA gene, shotgun metagenomics reveals all genetic information from the gut microbial ecosystem. It provides us with more comprehensive and in-depth insights into the gut microbiota in a high-throughput manner. Due to the large amount of genetic information of the gut microbiome and limitation of the current sequencing capacity, deep sequencing with short reads is most commonly used in metagenomic studies. The raw data obtained from metagenomic sequencing is usually composed of tens of millions of 100–150 bp short reads per sample. The short reads are then ordered and merged into longer genetic fragments (contigs) using various strategies, such as a form of de Bruijin graph based on the k-mer frequency pattern of reads [25]. Recent advances in assembly algorithms and related methodologies have significantly improved the accuracy and efficiency of metagenomic assembly [26]. Individual genes identified via reads assembly and de novo prediction [27–29] are the basic units with biologically relevant information for assembly-based metagenomics. The non-redundant gut microbial gene catalog constructed in human metagenomic studies typically contains millions of genes from a few hundreds of samples, once again leading to the problem of high dimensionality and sparsity.

To reduce data dimensionality and sparsity, one often uses functional annotation of the predicted genes to aggregate individual genes into orthology groups, modules, and pathways incorporated in various tools [30–33] and applied in many studies [34–36]. This type of aggregation relies on existing databases to reduce dimensionality. For example, pathway analysis characterizes microbial functions based on pathway enrichment; it reduces data dimensionality and sparsity via assigning millions of individual genes into hundreds of pathways in a community-wide manner using databases such as KEGG [37] and MetaCyc [38], and subsequently identify the pathways that are relevant to host phenotypes. However, due to the limitation of current reference databases and annotation methods, many genes are annotated as unknown function/hypothetical proteins. For instance, 54% of the 17 million genes in KEGG cannot be annotated with KO (KEGG Orthology) number [39], and only 41.1% are annotated on KO and 60.4% annotated on eggNOG (evolutionary genealogy of genes: Non-supervised Orthologous Groups) in the IGC [40], an integrated catalog of reference genes in the human gut microbiome based on 1267 samples. Thus, analysis at the KO or eggNOG level could exclude around half of the genes. Overlooking these novel genes will lead to incomplete or spurious findings on the gut microbiome’s role in human health and diseases.

Gene-centric analysis also overlooks the important reality that genes contributing to the same function may come from different bacterial carriers. Thus, these genes may show conflicting patterns of abundance change because their respective bacterial carriers may have distinct growth trajectories within a given environment. For example, in our study of patients with type 2 diabetes [41], a high-fiber dietary intervention increased the gut microbial community’s capacity to produce butyrate, as evidenced by significantly higher fecal content of butyrate after 28 days of intervention. Associated with this increase was a significant increase in the community-wide abundance of the terminal gene *but* in the production pathway of butyrate. However, among the 30 prevalent bacterial strains that harbored the butyrate-producing pathway, only five strains were selectively promoted by the high-fiber diet, and these five were likely to be the main drivers of butyrate production. This finding suggests that strains possessing the same pathway may differ in their contribution to the community-wide expression of that pathway function. Gene-centric microbiome analysis overlooks this fact and may confound our understanding of gut microbiota-host interactions.

## Ecological guilds as units for microbiome data reduction

Members of an ecosystem seldomly live independently from each other; instead, they develop local interactions and form inter-member organizations to influence higher-level patterns and functions of the ecosystem [42]. In macro-ecology, an important form of inter-member organization is called “guild,” a term initially coined to represent “a group of species that exploit the same class of environmental resources in a similar way” [43]. Later, guilds became synonymous with “functional groups,” and members of the same guild also encompass those who perform similar functions within a community [43]. In this context of “guild” definitions, members of a guild tend to exhibit co-abundance patterns by thriving or declining together without regard to their taxonomic positions whenever resources become available or depleted.

Like macro-ecosystems, e.g., a rain forest [44], the human gut microbiome is a complex ecosystem, and patterns and functions of a complex ecosystem emerge from localized interactions among its individual components and inter-member organizations [42, 45]. Grouping bacterial members into potential guilds and studying individual organisms’ guild-level organization could help us understand the gut microbiota’s structural and functional relationships and highlight the individual components that are key to system processes and functions. We see a value in translating the concept of guild from macro-ecology to the study of microorganisms because guild focuses on interactions among individual members of an ecosystem without regard to their taxonomic positions. In addition, grouping bacterial members into guilds acknowledges the possibility of different species possessing potential molecular mechanisms that effectively “bind” them together to perform a specific functional role in the gut ecosystem. An example of a possible molecular mechanism is how one bacterium could produce a metabolic product used by another as a nutrient in a syntrophic relationship. Such a molecular mechanism could lead to these bacteria consistently increasing or decreasing in abundance and working together to fulfill certain metabolic functions or be more ecologically competitive. We propose that members of the same bacterial guild [46] are likely to work together to exert ecological functions and show consistent co-abundant behavior, which may be derived from exploiting the same resources in a similar way or possessing certain interactive molecular mechanisms.

The concept of guild has been used in various macro-ecological system studies, e.g., to explore the effects of environments on bird communities [47] or the assemblage of fish [48]. However, it is important to note that the definition of guild membership is sometimes arbitrary in macro-ecology because it is impossible to study all species living in an ecosystem at once, nor it is possible to monitor every member of a bird or fish community over an extensive period to capture their co-abundance patterns. In contrast, in the world of gut microbiota, one could capture data on every detectable member of the gut ecosystem, using high-throughput sequencing and along sizable spatial and temporal gradients, thus making it possible to statistically describe co-abundance patterns among all detectable members. This gives microbiome research the advantage to redefining guilds in the context of microbial ecosystems in a data-driven manner. The first step of partitioning potential guilds is to analyze bacterial abundance variations quantitatively. Whenever abundance data of genome markers (16S rRNA genes) or genomes are available, one could identify potential guilds by clustering co-abundance groups (CAGs) based on microorganisms’ co-variation of abundance, i.e., all members of the same potential guild (CAG) are positively correlated in the context of abundance changes. Consequently, clustering bacterial genomes into CAGs based on their abundance correlations could be the most parsimonious first step to study guild-level organization in microbiome research. The second step of studying guild-level organization is to investigate the relationships between guilds and host phenotypes. One effective strategy is to correlate guild abundance changes with variations of host bio-clinical parameters. The abundance of guilds can be represented by the sum of its members’ abundance since guild members have a concerted response to changes in the gut environment, e.g., increased availability of carbohydrates. Such correlation analysis can identify guilds into three categories: positively associated (potentially pathogenic), negatively associated (potentially beneficial), or have no association (potentially neutral) with disease phenotypes [46]. In this manner, guild-based analysis can potentially recapitulate the ecological interaction network of key members of the gut microbiota [49] and their relationship with host phenotypes.

Guild-based analysis can be an ecologically relevant aggregation method for identifying bacterial functional groups while reducing dimensionality and sparsity in data analysis. The concept of co-abundance group (CAG) was first introduced to the human microbiome field by Claesson et al. in 2012 [50]. Claesson and colleagues clustered correlated genera into six CAGs and investigated the transition of the gut microbiota, comparing between healthy community-dwelling subjects and frail long-term care residents. However, they clustered taxonomic groups instead of unique bacterial genomes and did not explore potential relationships between CAGs and host phenotypes. Since then, the concept of co-abundance group or co-abundance network has been applied in several dozens of microbiota studies [51–73]. Most of these studies [51–64], including Claesson et al. [50], first collapsed unique bacterial genomes into taxonomic units and then clustered taxonomic units into CAGs. The remaining publications [65–73] grouped unique genomes, operational taxonomic units (OTUs), or amplicon sequence variants (ASVs) into CAGs. However, these studies [65–73] did not treat CAGs as functional units nor directly investigated potential relationships between CAGs and host phenotypes. Though the concept of co-abundance analysis is not entirely new, our proposed strategy is different from previous work in two important aspects. First of all, we emphasize the importance of identifying CAGs at the highest resolution level possible (e.g., genome or strain in whole shotgun metagenome, OTU or ASV in 16S rRNA gene sequencing data), instead of analyzing co-abundant relationships at genus or higher taxon levels [50, 74]. Secondly, we treat CAGs as units with ecological functions (guilds) and directly explore their relationship with host phenotypes in down-stream analyses.

In practice, we suggest choosing suitable correlation methods based on specific dataset structures, such as the workflow proposed by Weisis et al. [75]. How to compare and select the best correlation methods for different microbiome datasets is beyond the scope of this article. However, in the next two sections of this opinion article, we present two guild-based analysis examples using two different correlation methods to demonstrate the possibility of integrating various correlation methods into guild-based analysis. Specifically, we recommend a dichotomic and tree-based group identification method for partitioning potential guilds without presetting the number of groups. In brief, the correlation coefficients between two genomes, two OTUs, or two ASVs could then be converted into distance metrics (1- correlation coefficient) and clustered using the Ward clustering algorithm [76]. From the top of the clustering tree, permutational multivariate analysis of variance (PERMANOVA test) is used to sequentially determine whether any of the two clades are significantly different [50]. For example, if the clustering tree has two big clades (A and B) and each one has two small clades (C and D belonging to A, and E and F belonging to B), PERMANOVA will be used to test the two larger clades first. If they are significantly different, one can test the two smaller clades under each big clade separately. If there is no significant difference between the smaller clades (C vs. D, E vs. F), the clustering will stop here and conclude that there are two potential guilds (A and B). The method we described above only serves as one example of clustering stop. Many conditions can be adopted to optimize the clustering results. One essential issue to note is that a minimum of 25 samples is needed for a robust co-abundance network analysis in microbiota studies [49]. Based on in silico stimulation work, Berry and Widder proposed several recommendations for applying co-abundance network analysis in microbiota studies [49]. A larger sample size (> 25 samples) will increase sensitivity for identifying more robust co-occurrence events. Lastly, we acknowledge that several other algorithms, such as autoencoding neural nets [77], and algorithms based on Singular Value Decomposition (SVD) [78], could also be used to reduce the dimensionality. However, whether these mathematical methods reflect the biological reality of the microbiome community remains elusive. These methods often reconstruct the dataset variables into new analysis features, such as encoder layers or principal components, making it more challenging to interpret the original variables (in this case, the individual bacterial strains). And finally, various traditional (e.g., model-based) and modern clustering (e.g., spectral graph theory based) algorithm [79] are also worth testing for the identification of guilds in future works.

### Guild-based analysis for metagenomic datasets

We first applied the concept of guild in a clinical trial to understand the role of gut microbiota in body weight regulation of obese children [46]. Genetically obese children with Prader-Willi syndrome (PWS) lost a significant amount of weight by consuming a diet enriched with non-digestible carbohydrates for 90 days. Fecal samples were collected at four time points (days 0, 30, 60, and 90) to track the associated gut microbiota changes [46] and deep metagenomic sequencing was performed on a total of 109 time series fecal samples (including two time point samples from a group of obese children with no genetic cause within the same intervention). 76.0 ± 18.0 million (mean ± s.d.) high-quality reads were obtained from each sample using the Illumina HiSeq platform. After de novo assembly with IDBA-UD [80], gene prediction with MetaGeneMark [81], and gene de-redundancy with CD-HIT [82], we generated a non-redundant gut microbial gene catalog containing ~ 2 million genes. The gene abundance matrix had a sparsity at 79% with more than 2 million dimensions (variables) for only 109 samples.

**Fig. 1:**
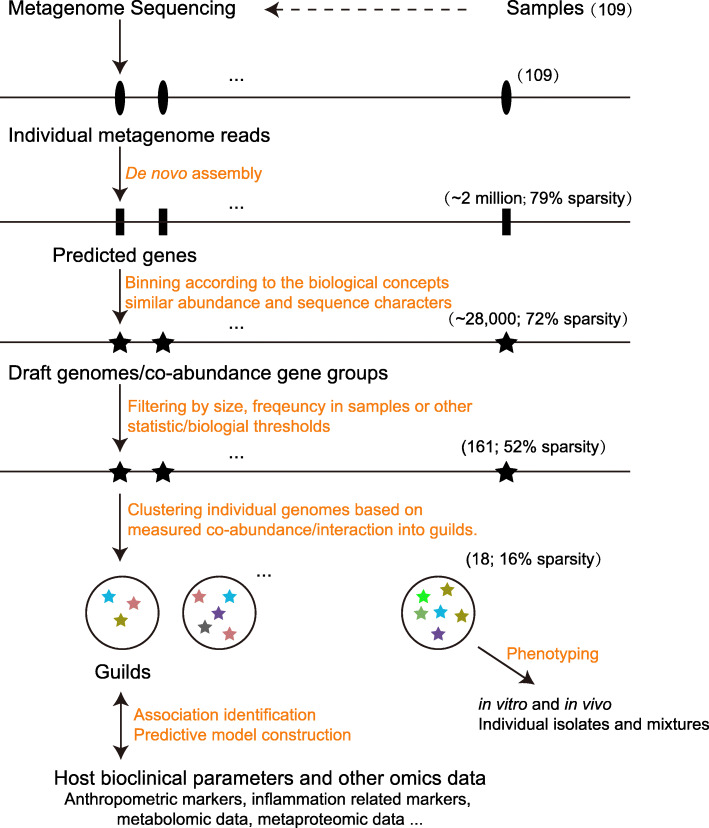
The reduction of dimensionality and sparsity from raw metagenomic dataset to genes, genomes, and guilds. In our PWS example, ~ 2 million non-redundant microbial genes were predicted from the 109 metagenomes. Seventy-nine percent of values in the corresponding abundance matrix of these genes were zeros. These non-redundant microbial genes were further binned into ~ 28,000 draft genomes based on their abundance correlations across the 109 samples. In the corresponding abundance matrix of these draft genomes, 72% of values were zeros. We then selected 161 prevalent bacterial genomes, each with more than 700 bacterial genes and shared by more than 20% of the samples. In the corresponding abundance matrix of these 161 genomes, 52% of values were zeros. Eighteen guilds were identified by clustering these prevalent bacterial genomes. In the corresponding abundance matrix of these 18 guilds, 16% values were zeros

Instead of searching the existing database for closest neighbors, we binned non-redundant genes into draft genomes based on the fact that the abundances of two genes located on the same genomic DNA molecule will highly correlate with each other across multiple samples [83]. A total of ~ 28,000 draft genomes (including both low-quality and high-quality ones) were binned and identified using a “canopy-based” algorithm [83]. Among all draft genomes, 376 had more than 700 genes and were identified as distinct bacterial genomes [83]. We further selected 161 genomes for subsequent analysis because they were shared by more than 20% of the samples and considered the prevalent gut bacteria. These selected genomes could be considered the predominant members of the gut microbiota because, together, they accounted for more than 60% of the total metagenomic sequences. At this point, we reduced the dataset to 161 variables with a sparsity of 52%. Then we calculated bootstrapped Spearman correlation coefficient to determine the associations between these 161 genomes. After converting the correlations into correlation distance, we clustered the 161 genomes into 18 guilds using the Ward clustering algorithm [76]. At this point, the guild abundance matrix was further reduced to a sparsity of 16%. Here we showed that a significant reduction of matrix dimensionality and sparsity is possible when we moved from genes to genomes and genomes to guilds (Fig. 1).

Our PWS study explored the relationship between these 18 identified guilds and disease phenotypes: three guilds showed negative correlations while nine guilds showed positive correlations with at least one disease phenotype. This result suggests that the former three guilds could potentially be beneficial, while the other nine guilds may be pathogenic and detrimental. The remaining six guilds had no correlations with any host disease phenotypes, indicating that they might be commensals. In addition, after reducing the data dimensionality, the number of variables was smaller than the sample size, allowing us to apply conventional/classical statistical models for modeling and predictions. Using a linear mixed-effect model trained with the day 0 and day 30 datasets, we showed that the guilds at day 60 had a predicting capacity for the anthropometric markers at day 90 (*R*^2^ = 0.64, *P* = 0.0002 for body mass index; *R*^2^ = 0.43, *P* = 0.0054 for hip circumference; *R*^2^ = 0.55, *P* = 0.0010 for waist circumference).

**Fig. 2:**
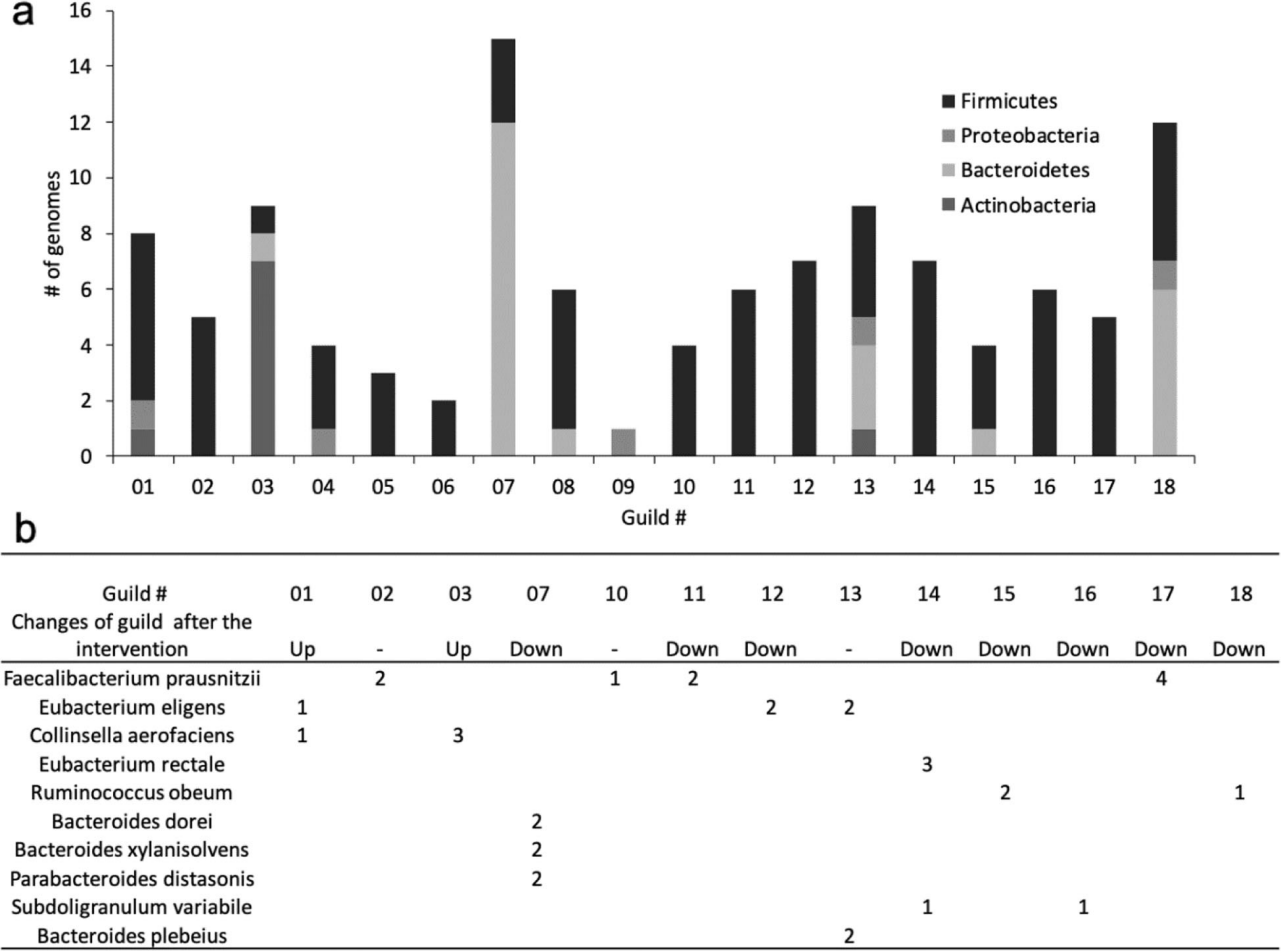
The taxonomic heterogeneity of guilds identified in the PWS study. **a** This stacked bar plot shows the phylum assignment of genomes belonging to each guild. **b** This table presents the distribution of species across guilds. The numbers in the table represent the number of genomes belonging to each species found in each guild. For example, 5 different genomes of the *Eubacterium eligens* species were found in guild#1, guild#12, and guild#13. A blank entry means that no genome from this species was found in this guild. “Up” denotes the guilds increased after the intervention, while “Down” indicates the guilds decreased after the intervention

**Fig. 3:**
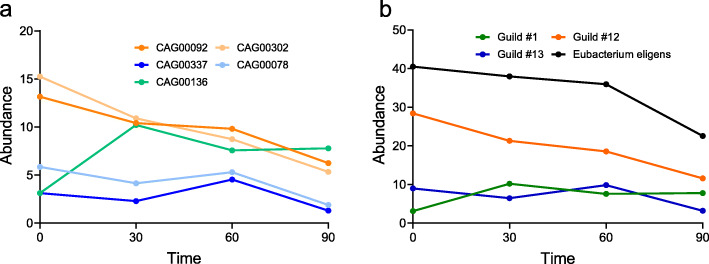
Guild-based aggregation overcomes the pitfall of taxon-based analysis to reflect the variations in strain-specific responses. **a** and **b** together illustrate why guild-based aggregation method produces a more accurate representation of strain-level microbiome response to dietary intervention in a PWS study compared with taxon-based aggregation. **a** shows the abundance change of the 5 *Eubacterium eligens* strains over time. If taxon-based aggregation is used, all 5 *Eubacterium eligens* strains could be collapsed into one species-level unit and represented by the black line in **b**. In contrast, using the guild-based aggregation method, the same 5 *Eubacterium eligens* strains are grouped into 3 different guilds (#1, #12, and #13). Each of the colored lines in **b** represents the abundance change over time of one guild. Abundance change pattern of the three guilds in **b** accurately captures the three types of abundance change patterns among the 5 *Eubacterium eligens* illustrated in **a***.* The dots on each line in **a** and **b** represent the mean abundance (see S.E.M in Supplementary Table 1)

Interestingly, in our PWS study, none of the guilds we identified was taxonomically homogeneous. The nine genomes in guild #13 were from four different phyla (Firmicutes, Proteobacteria, Bacteroidetes, and Actinobacteria; Fig. 2a). Meanwhile, bacteria that belonged to the same taxon (e.g., species) were assigned to different guilds that responded differently to the intervention. For example, five high-quality draft genomes of *Eubacterium eligens* were found in three different guilds: one positively responded, one negatively responded, and the third did not respond to the high-fiber intervention (Fig. 2b). Specifically, from day 0 to day 30, one *E. eligens* strain dramatically increased in abundance, while the other four showed a sharp or steady decrease (Fig. 3a). When these strains were collapsed at the species level, the abundance change of these five strains would have been added together and represented by the black line in Fig. 3b. Such a process would have overlooked the different response patterns of these *E. eligens* strains to the intervention, leading us to a spurious conclusion that the abundance of these *E. eligens* strains consistently decreased over the course of the intervention (Fig. 3b). This observation indicates that taxa are functionally heterogenous and do not serve as a coherent functional unit for correlation analysis with host phenotypes even at the species level. This result again echoes the limitations of taxon-based analysis, suggesting that bacterial taxa (species to phyla) are functionally heterogeneous units. By contrast, guild-based analysis groups the *E. eligens* strains into three guilds and accurately captures all three types of response patterns to the intervention (Fig. 3b).

### Guild-based analysis for 16S rRNA gene data

Guild-based analysis is also applicable for microbiome studies based on 16S rRNA gene sequencing. Conventionally, after quality control, reads are clustered into OTUs with 97% similarity using UPARSE [84] or other OTU picking methods. Recent advancement in the field is to perform denoising, which models and corrects sequencing amplicon errors, obtaining amplicon sequences variants (ASVs) with single-nucleotide resolution [85]. Studies can then identify guilds based on abundance correlations between OTUs/ASVs. Using a 16S rRNA gene V4 sequencing dataset from a study on green tea polyphenols, we found that the gut microbiome responded to polyphenols as guilds, and some guilds were associated with the polyphenols’ effect on lowering blood glucose in *db/db* mice [86]. In a series of studies on calorie restriction and gut microbiota, we applied the guild-based analysis to the 16S rRNA gene datasets. We identified a *Lactobacillus murinus*-dominated guild, which showed a positive correlation with lifespan and metabolic health and demonstrated a robust capacity to alleviate side-effects induced by a common chemotherapeutic agent [87–89].

In a study of patients with polycystic ovary syndrome (PCOS), 16S rRNA gene V3-V4 sequencing revealed the gut microbiome difference between PCOS patients and non-obese controls [90]. Out of 567 OTUs identified in total, 225 OTUs were shared by at least 20% of the samples and further clustered into 23 different guilds based on their co-abundance correlations calculated using the SparCC approach [91]. The abundance of these 23 guilds accounted for 95.53 ± 5.42% of the total sequences. The abundance of nine guilds showed a significant difference between patients and controls. When correlated with 26 host clinical parameters, three guilds (guilds #1, #4, and #7) showed positive correlations with disease parameters, and five (guilds #10, #11, #12, #13, and #18) were negatively correlated [90].

**Fig. 4:**
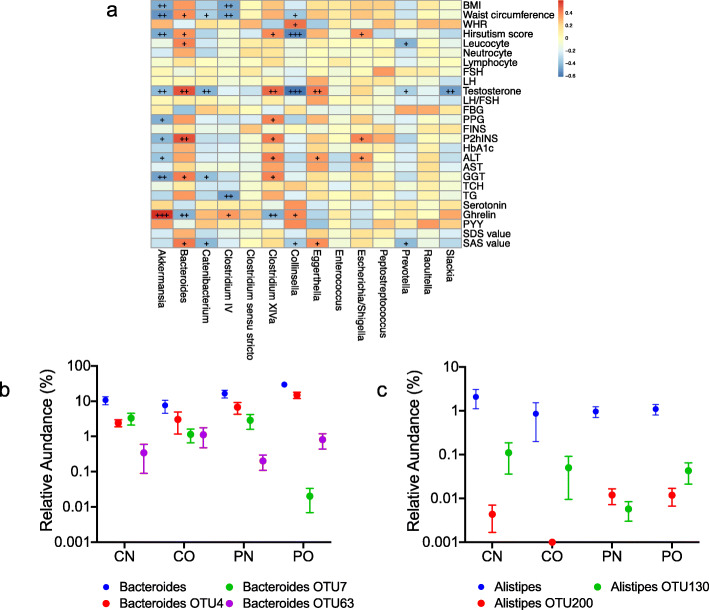
Comparing taxon-based and guild-based analysis in the PCOS study. **a** shows that the correlations between clinical parameters and prevalent genera are significantly different among PCOS patients and non-obese controls. The color of spots represents *R* value of the Spearman correlation between each genus and clinical parameter (+FDR < 0.05, ++FDR < 0.01, +++FDR < 0.001). **b** and **c** show the different abundance distributions of *Bacteroides* genus and 3 *Bacteroides* OTUs or *Alistipes* genus and *2 Alistipes* OTUs in different patient groups. *Bacteroides* OTU4 belonged to a guild that was positively correlated with disease phenotype, while *Bacteroides* OTU7 and *Bacteroides* OTU63 belonged to a negatively correlated guild. *Alistipes* OTU200 belonged to a guild that was positively correlated with disease phenotype, and *Alistipes* OTU130 belonged to a guild that was negatively correlated with disease phenotype. **a** For leucocyte, neutrocyte, lymphocyte, and hirsutism, *n* = 46; for the other parameters, *n* = 48. BMI, body mass index; WHR, waist hip ratio; FSH, follicular stimulating hormone; LH, luteinizing hormone; FPG, fasting plasma glucose; PPG, 2 h postprandial plasma glucose; FINS, fasting plasma insulin; P2hINS, 2 h postprandial plasma insulin; HbA1c, hemoglobin A1c; ALT, alanine aminotransferase; AST, aspartate transaminase; GGT, γ-glutamyltransferase; TCH, total cholesterol; TG, triglyceride; PYY, peptide YY; SDS, self-rating depression scale; SAS, self-rating anxiety scale. **b**, **c** CN, non-obese control group (*n* = 9); CO, obese control group (*n* = 6); PN, non-obese PCOS group (*n* = 12); PO, obese PCOS group (*n* = 21)

We performed taxon-based analysis at the genus level using the same OTU profile to demonstrate the difference between guild-based and taxon-based methods. Among the 567 OTUs, 266 OTUs were annotated into 96 genera [90]; 301 OTUs had no taxonomy annotation and were then excluded from subsequent analysis. We further narrowed the dataset to the 64 genera shared by more than 20% of the samples, a cut-off that was consistent with the guild-based analysis described above. These 64 genera, corresponding to 223 OTUs, accounted for 81.77 ± 7.5% of the total sequences. Fourteen genera showed a significant difference between the groups. Six genera had negative correlations with disease phenotypes, and four had positive correlations (Fig. 4a).

In this PCOS study, the taxon-based analysis excluded nearly 20% of raw sequencing data, an indication that a substantial part of the sequencing data was novel and had no close neighbor at the genus level in the reference database. Although using a similar number of OTUs as the taxon-based method, guild-based analysis kept novel sequencing data intact and did not restrict the analysis dataset to OTUs known in a reference database. Furthermore, guild-based analysis and taxon-based analysis of this PCOS dataset showed critical differences between their results on the potential role of specific OTUs in human health and disease phenotypes. First, there were discrepancies between results on *Bacteroides* from these two analysis strategies. A total of 13 prevalent OTUs were classified as *Bacteroides*. In taxon-based analysis, genus-level results showed positive correlations between *Bacteroides* genus and disease phenotypes (Fig. 4a), giving the impression that all OTUs in this genus may play a detrimental role in host health. Guild-based analysis clustered these 13 *Bacteroides* OTUs into seven different guilds. Specifically, *Bacteroides* OTU4 (classified into guild#1) showed a positive correlation with disease phenotype and were potentially detrimental, while *Bacteroides* OTU7 and *Bacteroides* OTU63 (both classified into guild#18) showed a negative correlation and were potentially beneficial (Fig. 4b). This result suggests that the assumed positive correlations between all OTUs in the genus *Bacteroides* and disease phenotypes, derived from taxon-based analysis results, could mislead our understanding of the roles of members of *Bacteroides* in PCOS. Secondly, when OTUs were collapsed at the genus level, the genus *Alistipes* did not correlate with host clinical parameters. In contrast, using guild-based analysis, we classified all eight prevalent *Alistipes* OTUs into five different guilds. Interestingly, *Alistipes* OTU130 was classified into guild#12, a guild that showed a negative correlation with host clinical parameters, while *Alistipes* OTU200 was classified into guild#4, a guild that was positively correlated with disease phenotypes (Fig. 4c). This finding suggests that different members of the same genus may affect the host in an opposite manner, some (as part of a guild) are positively associated with the disease phenotype, while others are negatively associated. When all the OTUs are collapsed into the same genus and used as a single variable in the analysis, OTUs with opposite relationships with the same disease phenotype could cancel each other out, resulting in a genus-level result of no correlation with the disease phenotype. Thirdly, only one OTU was annotated as *Akkermansia* at the genus level in the PCOS dataset. In the taxon-based analysis, this *Akkermansia* OTU80 had significantly different abundance among the groups and showed negative correlations with the host phenotype. In the guild-based analysis, the *Akkermansia* OTU80 was clustered into a guild, which also had significantly different abundance among the groups and showed negative correlations with the host phenotype. In addition to identifying *Akkermansia* as a potentially beneficial bacterium to host health, guild-based analysis revealed that *Akkermansia* OTU80 was co-abundant with one *Clostridium* IV OTU236 and another 8 OTUs without annotation at the genus level (unclassified OTU318, OTU272, OTU281, OTU397, OTU303, OTU300, OTU398, and OTU328). These findings suggest that the beneficial role of this *Akkermansia* OTU may require interactions with other bacteria [92]. Thus, guild-based analysis provides a complete picture of the ecological interactions between OTUs relevant to host health.

## Conclusions

The guild-based analysis we proposed here is a reference database independent and ecologically meaningful way for data aggregation that may lead to dissection of ecologically meaningful functional groups and identification of putative causative members of gut microbiota to specific disease phenotypes. This strategy overcomes two primary pitfalls of conventional taxon-based and gene-centric approach: (1) combining bacteria or genes that may have opposite relations with human health or diseases into new spurious variables and (2) focusing on bacteria or genes that are similar enough to those known in existing reference databases while ignoring the unknown and novel ones. Aggregating the microbial populations (strains) into guilds, or new variables that consider the ecological interactions between the microbiota members, will facilitate pattern recognition between microbiome and host phenotype or other metadata. It is pivotal for the microbiome research field to build new analytical methods or use existing methods, such as metabolic network reconstruction and modeling [93] or reverse ecology [94–96], to understand why and how guild members, especially those significantly correlated with host phenotypes, work together. Such investigation should eventually guide the isolation of bacterial strains as a consortium (functional groups) rather than single isolates. In mechanistic studies, these recognized patterns and isolates can help identify key functional gut bacteria contributing to human health and diseases causatively [97]. Guild-based analysis may create a paradigm shift towards an ecologically meaningful approach for understanding the relationship between the gut microbiome and human health.

## Supplementary Information

**Additional file 1: Supplementary Table 1.** The abundance of individual *Eubacterium eligens* strains, and that of the species and guilds presented in Fig. 3. The data were reported as Mean (S.E.M).

## Data Availability

All data analyzed as a part of this study is previously published.
