# Supplementary material for: Guild-based analysis for understanding gut microbiome in human health and diseases
Source: Genome Med. 2021 Feb 9;13:22. doi: 10.1186/s13073-021-00840-y (PMC7874449; doi:10.1186/s13073-021-00840-y)
Supplement: Supplementary file 1 — Additional file 1: Supplementary Table 1. The abundance of individual Eubacterium eligens strains, and that of the species and guilds presented in Fig. 3. The data were reported as Mean (S.E.M). [file 13073_2021_840_MOESM1_ESM.docx]

Supplementary Table 1. The abundance of individual *Eubacterium eligens* strains, and that of the species and guilds presented in Figure 3. The data were reported as Mean (S.E.M)

| Time | 0 | 30 | 60 | 90 |
| --- | --- | --- | --- | --- |
| CAG00078 | 5.85(2.66) | 4.15(1.65) | 5.3(3.35) | 1.9(0.95) |
| CAG00092 | 13.16(4.35) | 10.42(4.06) | 9.82(4.85) | 6.25(3.11) |
| CAG00136 | 3.13(2.48) | 10.21(4.5) | 7.57(4.46) | 7.78(3.5) |
| CAG00302 | 15.25(4.63) | 10.91(3.79) | 8.74(4.05) | 5.34(2.59) |
| CAG00337 | 3.13(1.15) | 2.3(0.88) | 4.53(3.27) | 1.31(0.68) |
| *Eubacterium.eligens* | 40.52(9.51) | 37.98(8.66) | 35.96(13.19) | 22.58(6.23) |
| Guild #1 | 3.13(2.48) | 10.21(4.5) | 7.57(4.46) | 7.78(3.5) |
| Guild #12 | 28.41(8.66) | 21.32(7.7) | 18.56(8.9) | 11.59(5.7) |
| Guild #13 | 8.98(3.79) | 6.44(2.52) | 9.84(6.61) | 3.21(1.6) |
